# Academic‐Year Differences in Dental Students' Knowledge of Non‐Ionising Imaging: MRI‐Related Dental Material Safety and Ultrasonography

**DOI:** 10.1111/eje.70309

**Published:** 2026-09-18

**Authors:** Alperen Tekin, Bengisu Deveci, Gökhan Tekin

**Affiliations:** ^1^ Department of Oral and Maxillofacial Radiology, Faculty of Dentistry Istanbul Medeniyet University Istanbul Türkiye; ^2^ Private Practice Kastamonu Türkiye

**Keywords:** curriculum, dental education, dental materials, imaging literacy, magnetic resonance imaging, ultrasonography

## Abstract

**Introduction:**

Non‐ionising imaging is increasingly relevant to dental education, but students may not develop operational knowledge of magnetic resonance imaging (MRI)‐related dental material safety or dentomaxillofacial ultrasonography. This study examined academic‐year differences in these domains among clinical‐year dental students.

**Materials and Methods:**

Two anonymous, literature‐based questionnaires were analysed separately in this single‐centre cross‐sectional study. The final locked samples comprised 188 complete MRI questionnaires (third/fourth/fifth year: 60/85/43) and 188 complete ultrasonography questionnaires (62/84/42); the anonymous forms could not be linked at the individual level. MRI items assessed theory and material‐specific artefact, heating, and movement/instability judgements. Ultrasonography items assessed indications, limitations, and advantages. Between‐year comparisons used Kruskal‐Wallis tests with Bonferroni‐adjusted post hoc analyses.

**Results:**

MRI total scores differed across years (16.98 ± 10.46, 21.61 ± 9.16 and 21.72 ± 8.79; *p* = 0.017), mainly through theory items (*p* < 0.001) and heat‐generation judgements (*p* = 0.005). Artefact and movement/instability subscores did not differ significantly. Ultrasonography knowledge increased more consistently (14.29 ± 7.46, 17.36 ± 6.23 and 19.33 ± 4.46; *p* = 0.001), with significant differences in indications, limitations and advantages.

**Conclusion:**

Academic‐year differences were observed, but misconceptions persisted in both imaging domains. Undergraduate curricula should incorporate structured, case‐based non‐ionising imaging literacy training that distinguishes MRI image‐quality effects from safety risks and clarifies context‐dependent uses of ultrasonography.

**Abbreviations**
AP: artefact production
BMI: body mass index
CBCT: cone‐beam computed tomography
HG: heat generation
IDTV: instability due to vibration
IQR: interquartile range
MRI: magnetic resonance imaging
MV: movement/instability
TMJ: temporomandibular joint
US: ultrasonography

## 1 Introduction

Non‐ionising imaging is increasingly relevant to dental education because graduates must select modalities according to the diagnostic task, recognise important limitations and communicate appropriately with imaging services. Magnetic resonance imaging (MRI) and ultrasonography (US) complement conventional radiography and cone‐beam computed tomography (CBCT) when soft‐tissue or dynamic information is required. MRI is particularly useful for temporomandibular joint (TMJ) soft tissues, salivary glands and selected maxillofacial conditions, whereas US offers portable, real‐time assessment of superficial soft tissues, salivary glands, cervical lymph nodes and selected TMJ or jaw findings [1, 2, 3, 4, 5, 6, 7, 8].

For MRI, the key educational challenge is not simply recognising that intraoral materials can interact with the magnetic field. Dental restorations, implants and orthodontic components may affect image quality through susceptibility artefact and, depending on material and configuration, may also raise questions about heating or movement. These effects are not interchangeable and vary with composition, geometry, field strength, sequence and proximity to the diagnostic region [1, 9, 10, 11, 12, 13, 14, 15, 16, 17, 18, 19, 20]. Material‐specific reasoning is therefore preferable to the simplified assumption that any metal in the mouth represents the same MRI risk.

Ultrasonography presents a different knowledge problem. Its lack of ionising radiation, portability, repeatability and real‐time imaging make it attractive for several dentomaxillofacial applications, but its usefulness is highly context dependent. Operator dependence and acoustic restriction from bone or air remain important limitations, and US may be adjunctive rather than definitive for intraosseous lesions or TMJ assessment [2, 3, 4, 5, 6, 8, 21, 22, 23]. Appropriate use therefore requires students to understand both where US can contribute and where MRI, CT or CBCT remain preferable.

Available educational studies suggest that these competencies are uneven. Dental professionals have shown incomplete knowledge of MRI‐related material interactions, particularly when artefact, heating and instability are considered separately [24, 25, 26, 27]. For US, students and practitioners generally report favourable attitudes but variable knowledge and limited formal exposure, supporting calls for more structured undergraduate teaching [7, 28]. Recent dental education surveys likewise show the value of mapping knowledge and curricular exposure at the cohort level to identify specific training needs [29, 30, 31].

Most previous surveys, however, have considered MRI‐related material knowledge and dentomaxillofacial US separately. Comparing academic years in the two domains can therefore help identify whether increasing clinical seniority is associated with more accurate knowledge and where misconceptions persist.

Accordingly, this study evaluated academic‐year differences among third‐, fourth‐ and fifth‐year dental students in (1) MRI‐related dental material interactions, including artefact production, heat generation and movement/instability, and (2) the indications, limitations and advantages of dentomaxillofacial US. The primary objective was to determine whether knowledge differed by academic year within each modality and to localise the main misconception patterns relevant to undergraduate teaching.

## 2 Materials and Methods

### 2.1 Study Design, Setting and Ethics

This cross‐sectional questionnaire study was conducted at a faculty of dentistry in Türkiye. The study protocol was reviewed and approved by the institutional non‐interventional health research ethics committee before participant recruitment. Participation was voluntary and anonymous. All students provided electronic informed consent before accessing the questionnaires, and no directly identifying personal data were collected.

The study is reported with reference to the STROBE recommendations for cross‐sectional observational studies. Because the project was an anonymous educational survey and did not involve an intervention or allocation of participants to health‐care treatments, trial registration was not applicable.

### 2.2 Participants and Eligibility

The target population comprised undergraduate dental students in the clinical phase of training (3rd, 4th and 5th years). The MRI and US questionnaires were distributed as separate modality‐specific anonymous forms during the same survey period. The study was designed to compare academic‐year performance within each modality rather than to perform paired MRI‐versus‐US comparisons. Consistent with the approved anonymous survey protocol, the forms were therefore administered independently and no cross‐form linkage identifier was collected. Completion of one questionnaire was not a condition for completing the other.

Consequently, some students may have completed both questionnaires, but the degree of overlap cannot be determined. Each dataset was therefore analysed separately as an unlinked survey sample, and no within‐student or paired cross‐modality comparisons were performed. Eligible students who consented and submitted complete questionnaires before the predefined data‐lock were included; incomplete or late responses were excluded, and no missing‐item imputation was used.

No formal a priori power calculation was used. The ethics protocol anticipated approximately 150–190 volunteer participants from the accessible clinical‐year population, and recruitment was open to eligible students during the predefined survey window. The final locked datasets each contained 188 complete questionnaires. Because the forms were anonymous and invitations were distributed at course level without individual tracking, a precise response rate against the full class roster could not be calculated. An ancillary post hoc achieved‐power analysis for the primary total‐score comparisons was conducted to contextualise the achieved sample size and is described below.

### 2.3 Questionnaire Development and Content Review

Both questionnaires were developed as criterion‐referenced educational knowledge assessments rather than psychometric scales intended to measure a single latent construct. Development proceeded from a literature‐based construct map: the MRI instrument included two general‐principle items and 48 material‐interaction judgements covering 16 dental materials across artefact, heating and movement/instability domains, while the US instrument mapped 28 items to indications, limitations and advantages.

The study team reviewed each item for clinical relevance, clarity, redundancy and alignment with the learning needs of clinical‐year dental students, and each item was mapped to the literature used for content interpretation and answer‐key construction [2, 3, 4, 5, 6, 7, 8, 9, 10, 11, 12, 13, 15, 16, 17, 18, 19, 20, 21, 22, 23, 24, 25, 26, 27, 28, 29, 30, 31, 32]. This was a qualitative content‐review process; a formal independent expert‐panel content validity index was not calculated. Because the items deliberately sampled heterogeneous clinical knowledge rather than a single latent trait, internal‐consistency statistics such as Cronbach's alpha were not used as primary validity evidence.

### 2.4 Ultrasonography Questionnaire

The US questionnaire comprised 28 knowledge items organised into three domains: indications (12 items), limitations (9 items), and advantages (7 items). Indication items were answered as yes, no, or do not know, whereas limitation and advantage items were rated as agree, disagree, or unsure.

The items targeted practical dentomaxillofacial scenarios, including odontogenic and non‐odontogenic cystic lesions, soft‐tissue tumours, salivary gland pathology, TMJ conditions, lymph node findings, maxillary sinus pathology, trauma, and pre‐implant soft‐tissue evaluation, together with core operational aspects such as operator dependence, portability, real‐time imaging, and the effects of bone and air on image acquisition. The US questionnaire structure was informed by major reviews and systematic reviews addressing oral and maxillofacial lesion assessment, TMJ imaging, salivary gland applications, periodontal and peri‐implant imaging, and broader dentomaxillofacial uses of US [2, 3, 4, 5, 6, 8, 21, 22, 23].

### 2.5 Answer‐Key Construction and Scoring Strategy

The answer keys used for the primary analyses were finalised before those analyses were run. For MRI, primary scoring used an article‐based, evidence‐weighted operational key anchored to foundational work by Mathew et al. and Hubálková et al. and interpreted using subsequent systematic reviews and material‐specific studies [9, 11, 12, 13, 15, 16, 17, 18, 19, 20]. The key was intended for research classification rather than patient‐level MRI clearance. Because reported interactions vary with material, geometry, field strength, sequence and region of interest, items with heterogeneous evidence were assigned the most defensible category after weighing the direction, consistency and clinical relevance of the available evidence.

Each MRI item was also assigned a qualitative confidence level (high, moderate or low), and a sensitivity analysis excluding low‐confidence items was prespecified. The two MRI principle items were summarised separately from the 48 material‐interaction items. Artefact, heat‐generation and movement/instability domains each ranged from 0 to 16; the interaction subtotal ranged from 0 to 48 and the total MRI score, including the two theory items, ranged from 0 to 50. Correct answers received one point; incorrect and do‐not‐know responses received zero points.

### 2.6 Scoring of the Ultrasonography Questionnaire

For the US questionnaire, the answer key was also defined a priori and reflected whether ultrasonography could be considered diagnostically helpful in the scenario described, rather than whether it represented the sole or reference‐standard examination in that setting [2, 3, 4, 5, 6, 8, 21, 22, 23]. This distinction was important for items such as intraosseous lesions, TMJ assessment, and certain preoperative applications, where US may function as an adjunct rather than a replacement technique. Each correct response received one point; incorrect and unsure responses received zero points. Domain scores were calculated for indications (0–12), limitations (0–9), and advantages (0–7), and these were summed to obtain a total US knowledge score ranging from 0 to 28.

### 2.7 Statistical Analysis

Data were tabulated and analysed using IBM SPSS Statistics, version 29.0 (IBM Corp., Armonk, NY, USA). Descriptive statistics were calculated for demographic variables, item‐level responses and domain‐level scores. Because the MRI and US questionnaires were analysed as separate unlinked survey samples, all inferential analyses were performed within modality; no paired cross‐modality comparisons were attempted. Categorical response distributions across academic years were evaluated using Pearson's chi‐square test or Fisher's exact test, as appropriate.

Because the knowledge scores were bounded discrete variables and did not consistently satisfy normality assumptions, between‐year comparisons were primarily performed using the Kruskal–Wallis test. When the omnibus test was significant, post hoc pairwise comparisons were conducted with Bonferroni adjustment. Means ± standard deviations, medians with interquartile ranges, exact *p* values and adjusted pairwise *p* values are reported where relevant. No imputation was performed because only complete questionnaires were included. Item‐level analyses were prespecified as exploratory; their *p* values were not adjusted for multiplicity and should be interpreted descriptively. A two‐sided alpha level of 0.05 was used throughout.

An ancillary post hoc achieved‐power calculation was performed for the primary total MRI and total US between‐year comparisons. Because the primary inferential tests remained Kruskal–Wallis tests, power was estimated with a fixed‐effects omnibus one‐way ANOVA approximation at alpha = 0.05, using Cohen's f derived from the observed year‐specific means and pooled within‐group standard deviation. This calculation was used only to contextualise the achieved sample size and did not replace or alter the non‐parametric analyses. The power calculations were implemented with statsmodels 0.14.6 in Python 3.13.

## 3 Results

After application of the predefined final data‐lock, 188 MRI questionnaires and 188 ultrasonography questionnaires were available for analysis. The surveys were administered as separate anonymous forms, so participant overlap between the two 188‐response samples cannot be determined. The year‐level distributions differed slightly: the MRI sample comprised 60 third‐year, 85 fourth‐year, and 43 fifth‐year responses, whereas the US sample comprised 62, 84, and 42, respectively. Sex distribution was balanced in both surveys (MRI: 89 women and 99 men; US: 90 women and 98 men). Age‐group distributions are summarised in Table 1.

**TABLE 1:** Participant characteristics of the MRI and ultrasonography survey samples.

Characteristic	Category	MRI, *n* (%)	US, *n* (%)
Class year	3	60 (31.9%)	62 (33.0%)
	4	85 (45.2%)	84 (44.7%)
	5	43 (22.9%)	42 (22.3%)
Gender	Female	89 (47.3%)	90 (47.9%)
	Male	99 (52.7%)	98 (52.1%)
Age group	≤ 20	19 (10.1%)	20 (10.6%)
	21	31 (16.5%)	32 (17.0%)
	22	42 (22.3%)	41 (21.8%)
	23	51 (27.1%)	52 (27.7%)
	24	25 (13.3%)	23 (12.2%)
	≥ 25	20 (10.6%)	20 (10.6%)

Descriptive score distributions and omnibus between‐year comparisons are presented in Tables 2 and 3, while detailed item‐level correctness rates are provided in Tables S1 and S2. Selected lowest‐correctness items are summarised in Table 4. The principal score patterns are visualised in Figures 1, 2, 3, 4.

**TABLE 2:** MRI knowledge scores by academic year and omnibus between‐year comparisons.

Outcome	Year 3 mean ± SD	Year 3 median [IQR]	Year 4 mean ± SD	Year 4 median [IQR]	Year 5 mean ± SD	Year 5 median [IQR]	*H*	*p*	Post hoc (Bonferroni)
Theory score (0–2)	1.08 ± 0.83	1.0 [0.0–2.0]	1.65 ± 0.59	2.0 [1.0–2.0]	1.58 ± 0.59	2.0 [1.0–2.0]	20.672	< 0.001	3 vs. 4 (p_adj < 0.001); 3 vs. 5 (p_adj = 0.006)
Artefact subscore (0–16)	6.48 ± 4.02	6.0 [3.0–9.2]	6.87 ± 3.27	7.0 [5.0–9.0]	7.74 ± 3.18	8.0 [6.0–10.0]	3.752	0.153	NS
Heat‐generation subscore (0–16)	4.15 ± 3.41	4.0 [0.0–7.0]	6.26 ± 3.74	7.0 [4.0–9.0]	5.65 ± 3.90	6.0 [2.0–9.0]	10.421	0.005	3 vs. 4 (p_adj = 0.004)
Movement/instability subscore (0–16)	5.27 ± 4.00	6.0 [1.0–9.0]	6.84 ± 4.08	7.0 [4.0–10.0]	6.74 ± 3.66	8.0 [4.5–9.0]	5.035	0.081	NS
Interaction subtotal (0–48)	15.90 ± 10.20	16.0 [8.0–25.2]	19.96 ± 9.14	21.0 [15.0–27.0]	20.14 ± 8.56	22.0 [14.5–27.0]	6.578	0.037	NS
Total MRI knowledge score (0–50)	16.98 ± 10.46	17.0 [8.0–27.2]	21.61 ± 9.16	23.0 [17.0–28.0]	21.72 ± 8.79	24.0 [16.0–28.5]	8.106	0.017	3 vs. 4 (p_adj = 0.030)

**TABLE 3:** Ultrasonography knowledge scores by academic year and omnibus between‐year comparisons.

Outcome	Year 3 mean ± SD	Year 3 median [IQR]	Year 4 mean ± SD	Year 4 median [IQR]	Year 5 mean ± SD	Year 5 median [IQR]	*H*	*p*	Post hoc (Bonferroni)
Indication subscore (0–12)	5.24 ± 3.81	6.0 [0.2–8.0]	6.68 ± 3.01	7.0 [5.0–9.0]	7.45 ± 2.35	8.0 [6.0–9.0]	8.231	0.016	3 vs. 5 (p_adj = 0.020)
Limitation subscore (0–9)	5.15 ± 2.52	5.0 [4.0–7.0]	6.00 ± 2.37	6.0 [5.0–8.0]	6.64 ± 1.91	7.0 [5.2–8.0]	10.594	0.005	3 vs. 5 (p_adj = 0.005)
Advantage subscore (0–7)	3.90 ± 2.32	5.0 [2.2–6.0]	4.68 ± 1.89	5.0 [4.0–6.0]	5.24 ± 1.34	6.0 [5.0–6.0]	9.321	0.009	3 vs. 5 (p_adj = 0.010)
Total US knowledge score (0–28)	14.29 ± 7.46	16.5 [9.2–20.0]	17.36 ± 6.23	19.0 [15.8–21.0]	19.33 ± 4.46	20.0 [18.0–22.0]	13.644	0.001	3 vs. 4 (p_adj = 0.036); 3 vs. 5 (p_adj = 0.001)

**TABLE 4:** Lowest‐correctness items identified in the MRI and ultrasonography questionnaires.

Survey	Item	Correct answer	Correct responses	Most common alternative response
MRI	Heating: Stainless steel	Risk absent	22/188 (11.7%)	Risk present (55.3%)
MRI	Heating: Co‐Cr	Risk absent	22/188 (11.7%)	Risk present (48.9%)
MRI	Heating: Ni‐Cr	Risk absent	25/188 (13.3%)	Risk present (48.4%)
MRI	Movement/instability: Metal orthodontic bracket	Risk absent	25/188 (13.3%)	Risk present (54.3%)
MRI	Movement/instability: Co‐Cr	Risk absent	29/188 (15.4%)	Risk present (50.5%)
MRI	Artefact production: Amalgam	No/negligible artefact	29/188 (15.4%)	Significant artefact (44.7%)
MRI	Heating: Prosthetic magnetic keeper	Risk absent	30/188 (16.0%)	Risk present (42.6%)
MRI	Movement/instability: Stainless steel	Risk absent	32/188 (17.0%)	Risk present (50.0%)
MRI	Movement/instability: Ni‐Cr	Risk absent	34/188 (18.1%)	Risk present (44.1%)
MRI	Movement/instability: Metal/cast post	Risk absent	34/188 (18.1%)	Risk present (48.4%)
US	Advantage: It enables assessment of intraosseous lesions.	Agree	46/188 (24.5%)	Disagree (64.4%)
US	Indication: Trauma patients	Yes	63/188 (33.5%)	No (36.2%)
US	Indication: Odontogenic cystic lesions	Yes	71/188 (37.8%)	No (35.6%)
US	Indication: Maxillary sinus pathologies	Yes	73/188 (38.8%)	No (33.5%)
US	Indication: Non‐odontogenic cystic lesions	Yes	77/188 (41.0%)	No (32.4%)
US	Indication: TMJ disc deformities	Yes	88/188 (46.8%)	Do not know (27.7%)
US	Limitation: Beard/moustache presence makes imaging difficult in male patients.	Agree	89/188 (47.3%)	Disagree (39.4%)
US	Indication: Trigeminal neuralgia	No	93/188 (49.5%)	Do not know (35.6%)
US	Limitation: It requires a special room.	Disagree	99/188 (52.7%)	Agree (36.7%)
US	Indication: TMJ bone deformities	No	100/188 (53.2%)	Do not know (29.3%)

**FIGURE 1:**
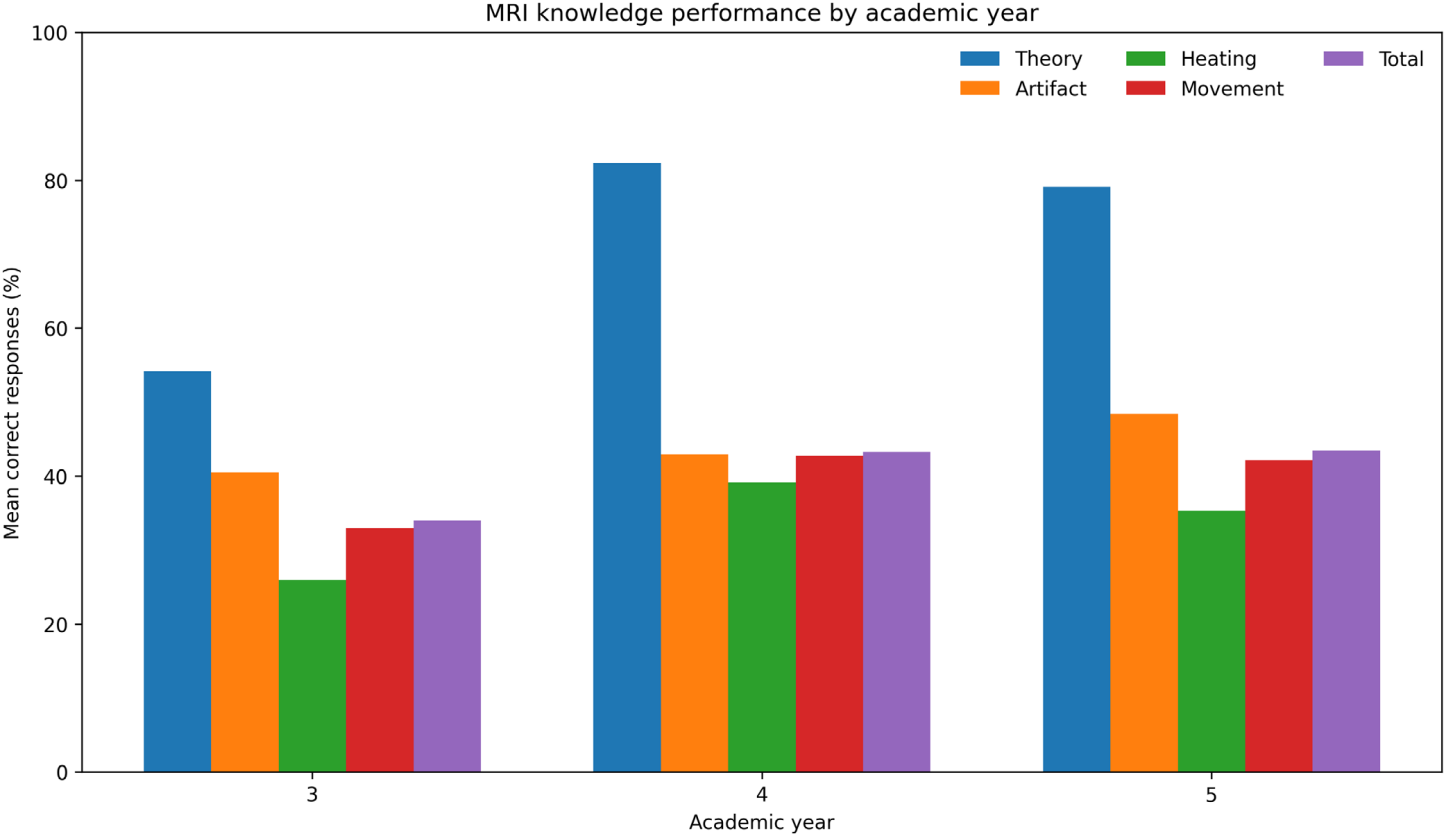
MRI knowledge performance by academic year. Bars represent mean percentages of correct responses for theory, artefact, heating, movement/instability, and total MRI scores.

### 3.1 MRI Questionnaire Findings

Overall MRI performance was modest. The mean total MRI knowledge score was 20.16 ± 9.71 out of 50, corresponding to 40.3% correct responses. By domain, the highest average performance was observed for artefact items (mean 6.95 ± 3.52/16), followed by movement/instability items (6.31 ± 4.01/16) and heat‐generation items (5.45 ± 3.77/16).

Total MRI knowledge differed significantly across academic years (Kruskal–Wallis *H* = 8.106, *p* = 0.017; Table 2; Figure 1). Third‐year students had lower total scores than fourth‐year students (median 17.0 [IQR 8.0–27.3] vs. 23.0 [17.0–28.0]; Bonferroni‐adjusted *p* = 0.030), whereas the third‐ versus fifth‐year comparison did not remain significant after adjustment (adjusted *p* = 0.062), and no difference was observed between the fourth and fifth years (adjusted *p* = 1.000).

This pattern was driven mainly by the theory items and the heat‐generation domain. The theory score differed significantly across years (*H* = 20.672, *p* < 0.001), with third‐year students scoring lower than both fourth‐year (adjusted *p* < 0.001) and fifth‐year students (adjusted *p* = 0.006). Heat‐generation scores also differed significantly (*H* = 10.421, *p* = 0.005), with a significant pairwise difference between the third and fourth years (adjusted *p* = 0.004). In contrast, artefact production (*p* = 0.153) and movement/instability (*p* = 0.081) subscores did not show statistically significant between‐year differences (Table 2).

Item‐level analysis revealed persistent material‐specific misconceptions (Table S1; Figure 3). The lowest correct‐response rates were observed for heat‐generation items involving stainless steel (11.7%), Co‐Cr (11.7%), and Ni‐Cr (13.3%), for which the most common response was risk present rather than the evidence‐weighted key response of risk absent. Movement/instability risk was likewise over‐attributed to metal orthodontic brackets (13.3% correct), Co‐Cr (15.4%), stainless steel (17.0%), Ni‐Cr (18.1%), and metal/cast posts (18.1%) (Table 4). Within the artefact domain, amalgam was frequently overclassified as producing marked artefact; only 15.4% selected the key response of no/negligible artefact, whereas 44.7% classified it as significant artefact.

Exploratory item‐level between‐year comparisons showed scattered rather than uniform progression (Table S1). Significant unadjusted between‐year variation was observed for artefact classification of Co‐Cr, acrylic resin, and metal orthodontic brackets, and for several heating and movement/instability items, including amalgam Co‐Cr, Ni‐Cr, metal‐ceramic restorations, metal/cast posts, and metal orthodontic brackets (all *p* < 0.05). Given the exploratory nature and multiplicity of these comparisons, these item‐level differences should be interpreted as supportive patterns rather than as the primary inferential basis of the study. However, the overall pattern remained one of persistent misconceptions across all three clinical years.

A sensitivity analysis excluding low‐confidence MRI items yielded the same directional pattern for the total score (*H* = 7.494, *p* = 0.024), although none of the Bonferroni‐adjusted pairwise comparisons remained statistically significant. This suggests that the principal MRI findings were not driven solely by the most uncertain answer‐key items.

For the primary total MRI score comparison, the ancillary post hoc analysis yielded Cohen's f = 0.229 and achieved power = 0.802.

### 3.2 Ultrasonography Questionnaire Findings

Because the two surveys were unlinked and used different score ranges, no inferential cross‐modality comparison was made. The mean total US knowledge score was 16.79 ± 6.59 out of 28, corresponding to 60.0% correct responses. Mean subscores were 6.38 ± 3.27/12 for indications, 5.86 ± 2.39/9 for limitations, and 4.55 ± 2.00/7 for advantages.

Total US knowledge increased significantly with academic year (*H* = 13.644, *p* = 0.001; Table 3; Figure 2). Mean ± SD total scores were 14.29 ± 7.46 in the third year, 17.36 ± 6.23 in the fourth year, and 19.33 ± 4.46 in the fifth year. Post hoc testing demonstrated that third‐year students scored lower than both fourth‐year (adjusted *p* = 0.036) and fifth‐year students (adjusted *p* = 0.001), whereas fourth‐ and fifth‐year students did not differ significantly (adjusted *p* = 0.296).

**FIGURE 2:**
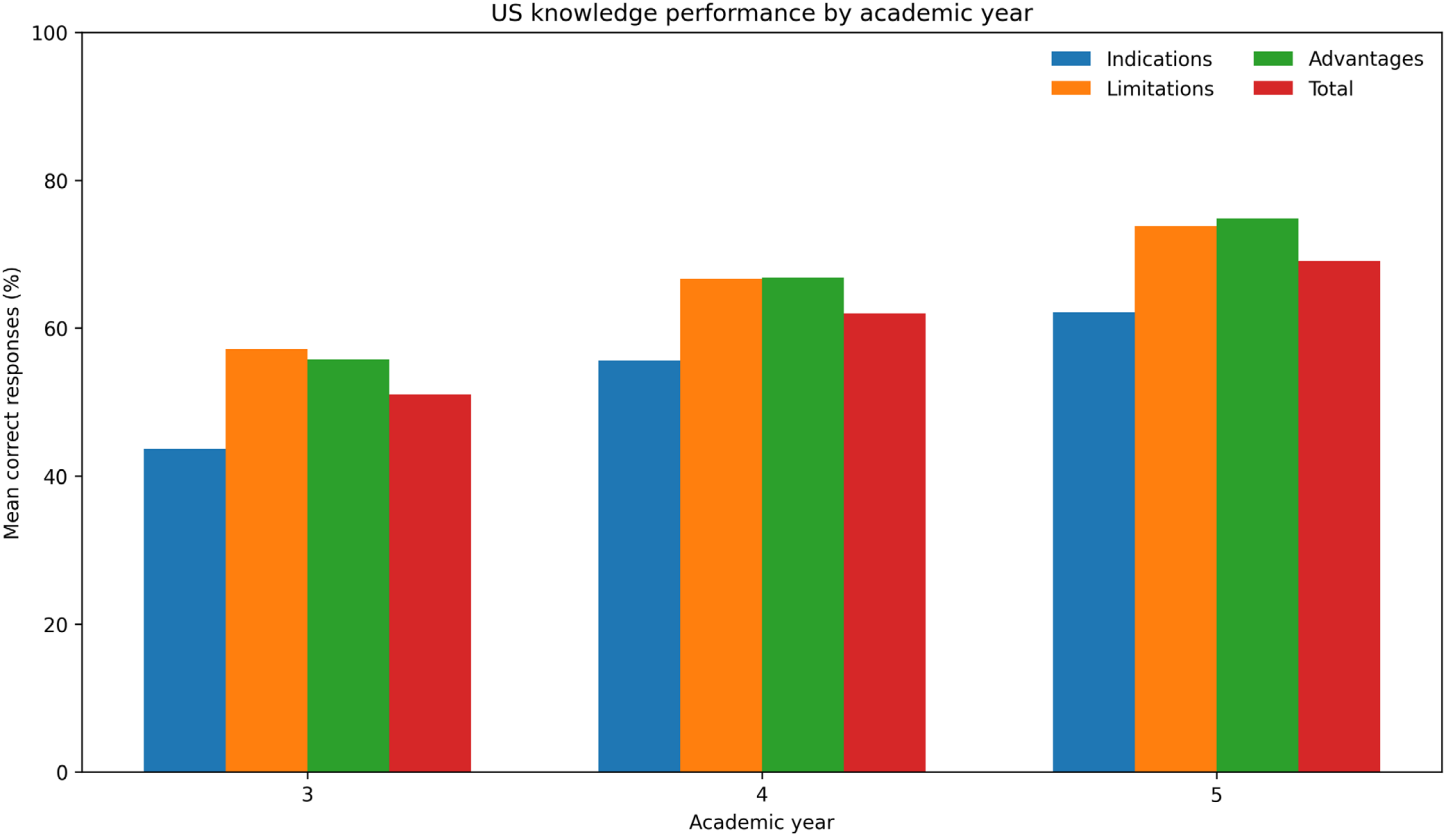
Ultrasonography knowledge performance by academic year. Bars represent mean percentages of correct responses for indication, limitation, advantage, and total US scores.

**FIGURE 3:**
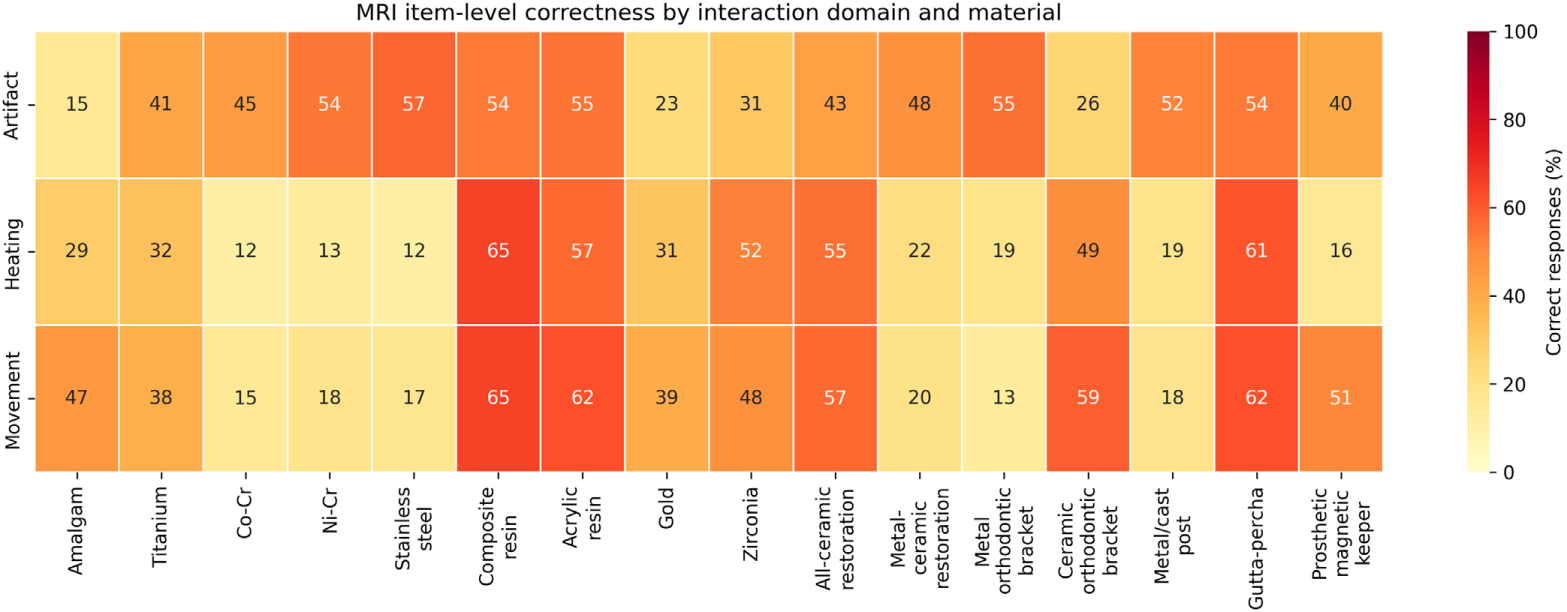
MRI item‐level correctness rates across materials and interaction domains. Rows represent interaction domains and columns represent dental materials; colours indicate the overall percentage of correct responses based on the evidence‐weighted answer key.

**FIGURE 4:**
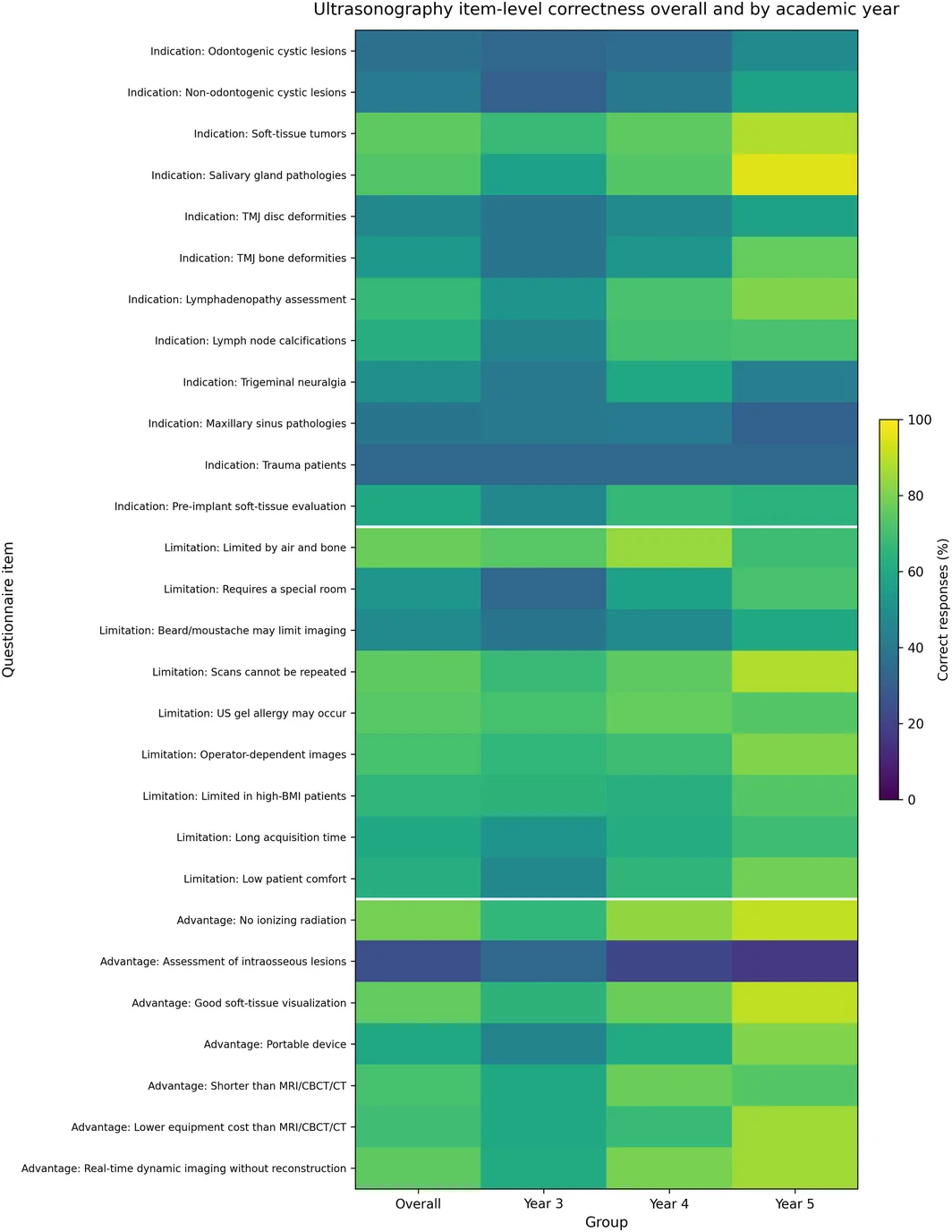
Ultrasonography item‐level correctness rates overall and by academic year. Rows represent questionnaire items grouped by domain and columns represent the overall cohort and academic years; colours indicate percentages of correct responses according to the revised answer key.

The same directional pattern was observed across all three US domains. Indication scores differed significantly across years (*H* = 8.231, *p* = 0.016), with a significant third‐ versus fifth‐year contrast after Bonferroni correction (adjusted *p* = 0.020). Limitation scores also differed significantly (*H* = 10.594, *p* = 0.005), again with a significant third‐ versus fifth‐year difference (adjusted *p* = 0.005). Advantage scores showed a comparable pattern (*H* = 9.321, *p* = 0.009), with higher fifth‐year than third‐year performance (adjusted *p* = 0.010) (Table 3).

The weakest US items concerned intraosseous lesion assessment, trauma indications, odontogenic and non‐odontogenic cystic lesions, maxillary sinus pathology, and TMJ disc deformities (Supplementary Table S2; Table 4; Figure 4). Only 24.5% of respondents correctly agreed that ultrasonography can enable assessment of intraosseous lesions in appropriate settings, and 64.4% incorrectly disagreed with this statement. Correct‐response rates were also low for trauma patients (33.5%), odontogenic cystic lesions (37.8%), maxillary sinus pathologies (38.8%), non‐odontogenic cystic lesions (41.0%), and TMJ disc deformities (46.8%). A substantial proportion of students also incorrectly agreed that US requires a special room (36.7% incorrect agreement).

Conversely, the strongest‐performing items were the absence of ionising radiation (79.3% correct), soft‐tissue visualisation (76.1%), real‐time/dynamic imaging (75.0%), salivary gland pathologies (72.9%), operator dependence (70.7%), lower equipment cost than MRI/CBCT/CT (69.1%), and lymphadenopathy assessment (67.0%) (Table S2). Exploratory item‐level comparisons showed significantly higher correctness in more senior students for salivary gland pathologies, TMJ bone deformities, lymphadenopathy, lymph node calcifications, portability, non‐ionising radiation, soft‐tissue visualisation, and the misconception that US requires a special room (all unadjusted *p* < 0.05; Table S2).

For the primary total US score comparison, the ancillary post hoc analysis yielded Cohen's f = 0.301 and achieved power = 0.965. Under the stated approximation, both achieved samples therefore provided approximately 80% or greater power for effects of the observed magnitude.

## 4 Discussion

This study found different academic‐year patterns across two non‐ionising imaging knowledge domains. MRI knowledge remained modest, with significant year differences in the total score driven mainly by theory and heating items, whereas artefact and movement/instability scores did not differ significantly. US knowledge showed a clearer seniority‐related increase across the total score and all three domains. The educational value of these findings lies in identifying the specific concepts that remained difficult rather than in making a direct quantitative comparison between the two unlinked surveys.

The most prominent MRI problem was failure to separate image‐quality effects from safety‐related interactions. Students frequently classified metallic materials as if artefact, heating and movement were interchangeable consequences, despite evidence that these effects depend on material composition, geometry, sequence, field strength and anatomic location [1, 9, 10, 11, 12, 13, 14, 15, 16, 17, 18, 19, 20]. This pattern is consistent with surveys showing incomplete professional awareness of MRI‐dental material interactions [24, 25, 26, 27]. Teaching that distinguishes ‘may degrade the image’ from ‘may create a clinically relevant safety concern’ could therefore address a concrete misconception rather than simply adding more general MRI content.

These findings refine earlier literature rather than merely repeat it: Bohner et al. showed that dental MRI artefacts vary with material and imaging conditions, while Gupta et al. reported incomplete professional knowledge of dental material‐MRI interactions [9, 25]. In our student sample, the educational gap was specifically the tendency to treat image degradation, heating and displacement as equivalent consequences.

The year pattern also suggests that conceptual exposure alone may not produce material‐specific reasoning. Theory scores improved most clearly, while artefact and movement/instability did not. A plausible educational explanation is that students encounter MRI mainly through indications such as TMJ or salivary‐gland imaging, with less practice applying MRI principles to restorations, implants, brackets, retainers, and wires. Case‐based exercises that require a material‐specific decision may therefore be more useful than repeating broad rules about metal and MRI.

Ultrasonography showed a more consistent progression with academic year, but several clinically important items remained weak. Students generally recognised core strengths such as absence of ionising radiation, soft‐tissue visualisation and real‐time imaging, yet many were uncertain about trauma, cystic lesions, maxillary sinus disease, intraosseous lesion assessment and TMJ applications. These topics are inherently qualified: US can be useful when anatomy provides an acoustic window or when a superficial or dynamic target is being assessed, but it does not universally replace MRI, CT or CBCT [2, 3, 4, 5, 6, 8]. The low performance on these items therefore appears to reflect difficulty with conditional indications rather than lack of awareness of US as a modality.

Weimer et al.'s call for sonography training is therefore supported here at the item level: deficits centred on conditional indications rather than basic advantages 7. This distinction is clinically meaningful because intraoral/intraoperative US has been studied for depth‐of‐invasion and margin assessment in oral tongue cancer, illustrating task‐specific value when an appropriate acoustic window is available [33, 34, 35, 36, 37].

Practical misconceptions were also evident. More than one third of students incorrectly believed that US requires a special room, despite portability being one of its established advantages [4, 5, 7, 8, 28]. Together with the context‐dependent indication errors, this suggests that undergraduate teaching should connect modality characteristics to actual referral scenarios rather than present indications and limitations as isolated lists.

Curricular implications should therefore remain targeted. For MRI, students should practise distinguishing artefact, heating and movement/instability and should consider material type, removability and the diagnostic region. For US, teaching should emphasise when the modality is a useful adjunct, the consequences of operator dependence and acoustic barriers, and when another modality remains preferable [1, 2, 3, 4, 5, 6, 8, 9]. This focused approach is consistent with calls for structured sonography training and with the broader use of needs‐assessment surveys in dental education [7, 28, 29, 30, 31].

Strengths of the study include literature‐derived scoring, domain‐ and item‐level analyses, and a sensitivity analysis for lower‐confidence MRI items. Several limitations affect interpretation. The study was conducted at a single Turkish dental school, so the observed year differences may reflect the sequencing, emphasis, and clinical exposure of one curriculum and may not generalise to institutions with different teaching models. The cross‐sectional design cannot distinguish curricular effects from cohort differences. The two questionnaires were administered as separate anonymous forms; participant overlap is unknown, preventing paired within‐student analyses or direct individual‐level cross‐modality comparison.

Although the instruments were built from published literature and underwent study‐team content review, no independent expert‐panel content validity index or formal psychometric validation was performed. Volunteer participation and the absence of individually tracked invitations may also introduce response or selection bias. Finally, some MRI key decisions required evidence‐weighted interpretation because universally standardised dental MRI guidance is limited; the sensitivity analysis was therefore retained. Multicentre, prospectively linked studies with formal content‐validation procedures would strengthen external validity and test whether targeted teaching changes these knowledge patterns.

Because these post hoc power estimates are calculated from observed effect sizes and are therefore closely related to the observed test results, they should be interpreted descriptively rather than as independent evidence of sample adequacy.

## 5 Conclusion

Knowledge differed by academic year, but the pattern varied by modality. MRI misconceptions clustered in material‐specific heating and movement/instability, whereas US knowledge improved more consistently with seniority but remained incomplete for several context‐dependent indications. Targeted case‐based teaching that separates MRI artefact from safety‐related effects and places US within its appropriate adjunctive indications and limitations may address these gaps.

## Author Contributions

A.T. contributed to the study conception and design, questionnaire framework, statistical analysis, interpretation of the results, manuscript drafting, preparation of tables and figures, and critical revision. B.D. contributed to study design, data collection coordination, data curation, literature review, and critical manuscript revision. G.T. contributed to questionnaire development, literature review, interpretation of the results, and critical manuscript revision. All authors reviewed and approved the final manuscript.

## Funding

The authors have nothing to report.

## Ethics Statement

The study was approved by the Istanbul Medeniyet University Faculty of Medicine Non‐Interventional Health Research Ethics Committee (protocol no: 2026‐GOSEK‐0101; meeting no: 4; decision no: 2026/04‐01; approval date: 29 April 2026). All participants provided electronic informed consent before completing the anonymous questionnaires.

## Conflicts of Interest

The authors declare no conflicts of interest.

## Supporting information


**Table S1:** MRI item‐level correct response rates according to the evidence‐weighted answer key.

**Table S2:** Ultrasonography item‐level correct response rates according to the revised answer key.

**Data S1:** STROBE Statement checklist for cross‐sectional studies.

## Data Availability

The data that support the findings of this study are available on request from the corresponding author. The data are not publicly available due to privacy or ethical restrictions.
