# Supplementary material for: Academic‐Year Differences in Dental Students' Knowledge of Non‐Ionising Imaging: MRI‐Related Dental Material Safety and Ultrasonography
Source: Eur J Dent Educ. 2026 Sep 18;30(4):1542–53. doi: 10.1111/eje.70309 (PMC13686681; doi:10.1111/eje.70309)
Supplement: Supplementary file 1 [file EJE-30-1542-s001.docx]

**Supplementary Table S1. MRI item-level correct response rates according to the evidence-weighted answer key.**

| **Item** | **Correct answer** | **Overall correct** | **Year 3** | **Year 4** | **Year 5** | **p** |
| --- | --- | --- | --- | --- | --- | --- |
| Artifact production: Amalgam | No/negligible artifact | 29/188 (15.4%) | 8/60 (13.3%) | 17/85 (20.0%) | 4/43 (9.3%) | 0.273 |
| Artifact production: Titanium | Minimal-to-moderate artifact | 78/188 (41.5%) | 25/60 (41.7%) | 39/85 (45.9%) | 14/43 (32.6%) | 0.352 |
| Artifact production: Co-Cr | Significant artifact | 85/188 (45.2%) | 31/60 (51.7%) | 30/85 (35.3%) | 24/43 (55.8%) | 0.043 |
| Artifact production: Ni-Cr | Significant artifact | 102/188 (54.3%) | 35/60 (58.3%) | 41/85 (48.2%) | 26/43 (60.5%) | 0.310 |
| Artifact production: Stainless steel | Significant artifact | 108/188 (57.4%) | 34/60 (56.7%) | 43/85 (50.6%) | 31/43 (72.1%) | 0.066 |
| Artifact production: Composite resin | No/negligible artifact | 101/188 (53.7%) | 29/60 (48.3%) | 47/85 (55.3%) | 25/43 (58.1%) | 0.595 |
| Artifact production: Acrylic resin | No/negligible artifact | 103/188 (54.8%) | 25/60 (41.7%) | 47/85 (55.3%) | 31/43 (72.1%) | 0.011 |
| Artifact production: Gold | No/negligible artifact | 44/188 (23.4%) | 12/60 (20.0%) | 24/85 (28.2%) | 8/43 (18.6%) | 0.403 |
| Artifact production: Zirconia | Minimal-to-moderate artifact | 58/188 (30.9%) | 20/60 (33.3%) | 25/85 (29.4%) | 13/43 (30.2%) | 0.900 |
| Artifact production: All-ceramic restoration | No/negligible artifact | 81/188 (43.1%) | 23/60 (38.3%) | 41/85 (48.2%) | 17/43 (39.5%) | 0.450 |
| Artifact production: Metal-ceramic restoration | Significant artifact | 91/188 (48.4%) | 26/60 (43.3%) | 41/85 (48.2%) | 24/43 (55.8%) | 0.466 |
| Artifact production: Metal orthodontic bracket | Significant artifact | 104/188 (55.3%) | 26/60 (43.3%) | 45/85 (52.9%) | 33/43 (76.7%) | 0.002 |
| Artifact production: Ceramic orthodontic bracket | Minimal-to-moderate artifact | 48/188 (25.5%) | 13/60 (21.7%) | 22/85 (25.9%) | 13/43 (30.2%) | 0.617 |
| Artifact production: Metal/cast post | Significant artifact | 97/188 (51.6%) | 29/60 (48.3%) | 42/85 (49.4%) | 26/43 (60.5%) | 0.425 |
| Artifact production: Gutta-percha | No/negligible artifact | 101/188 (53.7%) | 28/60 (46.7%) | 49/85 (57.6%) | 24/43 (55.8%) | 0.428 |
| Artifact production: Prosthetic magnetic keeper | Significant artifact | 76/188 (40.4%) | 25/60 (41.7%) | 31/85 (36.5%) | 20/43 (46.5%) | 0.544 |
| Heating: Amalgam | Risk absent | 55/188 (29.3%) | 9/60 (15.0%) | 31/85 (36.5%) | 15/43 (34.9%) | 0.013 |
| Heating: Titanium | Risk absent | 61/188 (32.4%) | 14/60 (23.3%) | 29/85 (34.1%) | 18/43 (41.9%) | 0.131 |
| Heating: Co-Cr | Risk absent | 22/188 (11.7%) | 2/60 (3.3%) | 15/85 (17.6%) | 5/43 (11.6%) | 0.022 |
| Heating: Ni-Cr | Risk absent | 25/188 (13.3%) | 3/60 (5.0%) | 18/85 (21.2%) | 4/43 (9.3%) | 0.015 |
| Heating: Stainless steel | Risk absent | 22/188 (11.7%) | 5/60 (8.3%) | 12/85 (14.1%) | 5/43 (11.6%) | 0.615 |
| Heating: Composite resin | Risk absent | 123/188 (65.4%) | 32/60 (53.3%) | 63/85 (74.1%) | 28/43 (65.1%) | 0.036 |
| Heating: Acrylic resin | Risk absent | 107/188 (56.9%) | 27/60 (45.0%) | 52/85 (61.2%) | 28/43 (65.1%) | 0.070 |
| Heating: Gold | Risk absent | 59/188 (31.4%) | 21/60 (35.0%) | 22/85 (25.9%) | 16/43 (37.2%) | 0.314 |
| Heating: Zirconia | Risk absent | 98/188 (52.1%) | 27/60 (45.0%) | 45/85 (52.9%) | 26/43 (60.5%) | 0.313 |
| Heating: All-ceramic restoration | Risk absent | 104/188 (55.3%) | 29/60 (48.3%) | 52/85 (61.2%) | 23/43 (53.5%) | 0.290 |
| Heating: Metal-ceramic restoration | Risk absent | 41/188 (21.8%) | 5/60 (8.3%) | 26/85 (30.6%) | 10/43 (23.3%) | 0.004 |
| Heating: Metal orthodontic bracket | Risk absent | 35/188 (18.6%) | 10/60 (16.7%) | 20/85 (23.5%) | 5/43 (11.6%) | 0.260 |
| Heating: Ceramic orthodontic bracket | Risk absent | 92/188 (48.9%) | 21/60 (35.0%) | 49/85 (57.6%) | 22/43 (51.2%) | 0.026 |
| Heating: Metal/cast post | Risk absent | 35/188 (18.6%) | 5/60 (8.3%) | 23/85 (27.1%) | 7/43 (16.3%) | 0.015 |
| Heating: Gutta-percha | Risk absent | 115/188 (61.2%) | 33/60 (55.0%) | 57/85 (67.1%) | 25/43 (58.1%) | 0.301 |
| Heating: Prosthetic magnetic keeper | Risk absent | 30/188 (16.0%) | 6/60 (10.0%) | 18/85 (21.2%) | 6/43 (14.0%) | 0.204 |
| Movement/instability: Amalgam | Risk absent | 88/188 (46.8%) | 25/60 (41.7%) | 43/85 (50.6%) | 20/43 (46.5%) | 0.571 |
| Movement/instability: Titanium | Risk absent | 72/188 (38.3%) | 17/60 (28.3%) | 37/85 (43.5%) | 18/43 (41.9%) | 0.162 |
| Movement/instability: Co-Cr | Risk absent | 29/188 (15.4%) | 3/60 (5.0%) | 20/85 (23.5%) | 6/43 (14.0%) | 0.007 |
| Movement/instability: Ni-Cr | Risk absent | 34/188 (18.1%) | 10/60 (16.7%) | 18/85 (21.2%) | 6/43 (14.0%) | 0.613 |
| Movement/instability: Stainless steel | Risk absent | 32/188 (17.0%) | 7/60 (11.7%) | 18/85 (21.2%) | 7/43 (16.3%) | 0.341 |
| Movement/instability: Composite resin | Risk absent | 123/188 (65.4%) | 35/60 (58.3%) | 57/85 (67.1%) | 31/43 (72.1%) | 0.319 |
| Movement/instability: Acrylic resin | Risk absent | 117/188 (62.2%) | 32/60 (53.3%) | 53/85 (62.4%) | 32/43 (74.4%) | 0.096 |
| Movement/instability: Gold | Risk absent | 74/188 (39.4%) | 21/60 (35.0%) | 34/85 (40.0%) | 19/43 (44.2%) | 0.636 |
| Movement/instability: Zirconia | Risk absent | 91/188 (48.4%) | 26/60 (43.3%) | 40/85 (47.1%) | 25/43 (58.1%) | 0.318 |
| Movement/instability: All-ceramic restoration | Risk absent | 107/188 (56.9%) | 31/60 (51.7%) | 50/85 (58.8%) | 26/43 (60.5%) | 0.606 |
| Movement/instability: Metal-ceramic restoration | Risk absent | 37/188 (19.7%) | 10/60 (16.7%) | 19/85 (22.4%) | 8/43 (18.6%) | 0.710 |
| Movement/instability: Metal orthodontic bracket | Risk absent | 25/188 (13.3%) | 2/60 (3.3%) | 17/85 (20.0%) | 6/43 (14.0%) | 0.008 |
| Movement/instability: Ceramic orthodontic bracket | Risk absent | 111/188 (59.0%) | 33/60 (55.0%) | 51/85 (60.0%) | 27/43 (62.8%) | 0.752 |
| Movement/instability: Metal/cast post | Risk absent | 34/188 (18.1%) | 7/60 (11.7%) | 19/85 (22.4%) | 8/43 (18.6%) | 0.269 |
| Movement/instability: Gutta-percha | Risk absent | 117/188 (62.2%) | 32/60 (53.3%) | 59/85 (69.4%) | 26/43 (60.5%) | 0.135 |
| Movement/instability: Prosthetic magnetic keeper | Risk present | 96/188 (51.1%) | 25/60 (41.7%) | 46/85 (54.1%) | 25/43 (58.1%) | 0.193 |
