# Supplementary material for: Academic‐Year Differences in Dental Students' Knowledge of Non‐Ionising Imaging: MRI‐Related Dental Material Safety and Ultrasonography
Source: Eur J Dent Educ. 2026 Sep 18;30(4):1542–53. doi: 10.1111/eje.70309 (PMC13686681; doi:10.1111/eje.70309)
Supplement: Supplementary file 2 [file EJE-30-1542-s002.docx]

**Supplementary Table S2. Ultrasonography item-level correct response rates according to the revised answer key.**

| **Item** | **Correct answer** | **Overall correct** | **Year 3** | **Year 4** | **Year 5** | **p** |
| --- | --- | --- | --- | --- | --- | --- |
| Indication: Odontogenic cystic lesions | Yes | 71/188 (37.8%) | 21/62 (33.9%) | 30/84 (35.7%) | 20/42 (47.6%) | 0.328 |
| Indication: Non-odontogenic cystic lesions | Yes | 77/188 (41.0%) | 19/62 (30.6%) | 34/84 (40.5%) | 24/42 (57.1%) | 0.030 |
| Indication: Soft-tissue tumors | Yes | 142/188 (75.5%) | 42/62 (67.7%) | 63/84 (75.0%) | 37/42 (88.1%) | 0.053 |
| Indication: Salivary gland pathologies | Yes | 137/188 (72.9%) | 35/62 (56.5%) | 62/84 (73.8%) | 40/42 (95.2%) | <0.001 |
| Indication: TMJ disc deformities | Yes | 88/188 (46.8%) | 24/62 (38.7%) | 40/84 (47.6%) | 24/42 (57.1%) | 0.196 |
| Indication: TMJ bone deformities | No | 100/188 (53.2%) | 24/62 (38.7%) | 44/84 (52.4%) | 32/42 (76.2%) | <0.001 |
| Indication: Lymphadenopathy assessment | Yes | 126/188 (67.0%) | 32/62 (51.6%) | 60/84 (71.4%) | 34/42 (81.0%) | 0.005 |
| Indication: Lymph node calcifications | Yes | 117/188 (62.2%) | 28/62 (45.2%) | 59/84 (70.2%) | 30/42 (71.4%) | 0.005 |
| Indication: Trigeminal neuralgia | No | 93/188 (49.5%) | 25/62 (40.3%) | 50/84 (59.5%) | 18/42 (42.9%) | 0.047 |
| Indication: Maxillary sinus pathologies | Yes | 73/188 (38.8%) | 25/62 (40.3%) | 35/84 (41.7%) | 13/42 (31.0%) | 0.508 |
| Indication: Trauma patients | Yes | 63/188 (33.5%) | 21/62 (33.9%) | 28/84 (33.3%) | 14/42 (33.3%) | 1.000 |
| Indication: Pre-implant soft-tissue evaluation | Yes | 112/188 (59.6%) | 29/62 (46.8%) | 56/84 (66.7%) | 27/42 (64.3%) | 0.040 |
| Limitation: Imaging is limited by air and bone. | Agree | 146/188 (77.7%) | 46/62 (74.2%) | 71/84 (84.5%) | 29/42 (69.0%) | 0.092 |
| Limitation: It requires a special room. | Disagree | 99/188 (52.7%) | 21/62 (33.9%) | 48/84 (57.1%) | 30/42 (71.4%) | <0.001 |
| Limitation: Beard/moustache presence makes imaging difficult in male patients. | Agree | 89/188 (47.3%) | 24/62 (38.7%) | 40/84 (47.6%) | 25/42 (59.5%) | 0.123 |
| Limitation: Scans cannot be repeated. | Disagree | 142/188 (75.5%) | 42/62 (67.7%) | 63/84 (75.0%) | 37/42 (88.1%) | 0.053 |
| Limitation: An allergic reaction to US gel may occur. | Agree | 139/188 (73.9%) | 44/62 (71.0%) | 64/84 (76.2%) | 31/42 (73.8%) | 0.790 |
| Limitation: Images are operator-dependent. | Agree | 133/188 (70.7%) | 41/62 (66.1%) | 58/84 (69.0%) | 34/42 (81.0%) | 0.241 |
| Limitation: Imaging is limited in patients with high BMI. | Agree | 124/188 (66.0%) | 40/62 (64.5%) | 53/84 (63.1%) | 31/42 (73.8%) | 0.487 |
| Limitation: Acquisition time is long. | Disagree | 113/188 (60.1%) | 32/62 (51.6%) | 52/84 (61.9%) | 29/42 (69.0%) | 0.191 |
| Limitation: Patient comfort is low. | Disagree | 117/188 (62.2%) | 29/62 (46.8%) | 55/84 (65.5%) | 33/42 (78.6%) | 0.002 |
| Advantage: It does not involve ionizing radiation. | Agree | 149/188 (79.3%) | 41/62 (66.1%) | 70/84 (83.3%) | 38/42 (90.5%) | 0.007 |
| Advantage: It enables assessment of intraosseous lesions. | Agree | 46/188 (24.5%) | 21/62 (33.9%) | 18/84 (21.4%) | 7/42 (16.7%) | 0.103 |
| Advantage: It visualizes soft-tissue lesions well. | Agree | 143/188 (76.1%) | 40/62 (64.5%) | 65/84 (77.4%) | 38/42 (90.5%) | 0.008 |
| Advantage: The device is portable. | Agree | 113/188 (60.1%) | 28/62 (45.2%) | 51/84 (60.7%) | 34/42 (81.0%) | 0.001 |
| Advantage: It provides shorter imaging time than MRI/CBCT/CT. | Agree | 133/188 (70.7%) | 37/62 (59.7%) | 65/84 (77.4%) | 31/42 (73.8%) | 0.068 |
| Advantage: It requires less expensive equipment than MRI/CBCT/CT. | Agree | 130/188 (69.1%) | 37/62 (59.7%) | 57/84 (67.9%) | 36/42 (85.7%) | 0.016 |
| Advantage: It provides real-time, dynamic imaging without reconstruction. | Agree | 141/188 (75.0%) | 38/62 (61.3%) | 67/84 (79.8%) | 36/42 (85.7%) | 0.008 |
