# Supplementary material for: Academic‐Year Differences in Dental Students' Knowledge of Non‐Ionising Imaging: MRI‐Related Dental Material Safety and Ultrasonography
Source: Eur J Dent Educ. 2026 Sep 18;30(4):1542–53. doi: 10.1111/eje.70309 (PMC13686681; doi:10.1111/eje.70309)
Supplement: Supplementary file 3 [file EJE-30-1542-s003.docx]

# STROBE Statement checklist for cross-sectional studies

Completed for: Academic-Year Differences in Dental Students’ Knowledge of Non-Ionizing Imaging: MRI-Related Dental Material Safety and Ultrasonography

| STROBE item | Where addressed |
| --- | --- |
| Title/abstract | Study design identified in the abstract; structured abstract provided. |
| Background/rationale | Scientific background and educational rationale described in the Introduction. |
| Objectives | Study objectives specified at the end of the Introduction. |
| Study design | Cross-sectional questionnaire design described in Methods. |
| Setting | Single faculty of dentistry in Türkiye; identifying institutional details are provided separately to the Editor and omitted here to preserve blinded review. |
| Participants | Eligibility criteria and clinical-year cohorts described in Methods. |
| Variables | MRI and ultrasonography knowledge domains and scores defined. |
| Data sources/measurement | Literature-based construct mapping, qualitative study-team content review, questionnaire domains, answer-key construction and scoring are described in Methods. |
| Bias | Voluntary participation, unlinked anonymous survey samples, single-centre design, possible selection/response bias and answer-key uncertainty are addressed in Methods/Limitations. |
| Study size | Protocol anticipated approximately 150-190 volunteers; achieved n=188 complete questionnaires per survey. No formal a priori power calculation; ancillary post hoc achieved power is reported. |
| Quantitative variables | Score ranges and item-level variables described. |
| Statistical methods | Kruskal-Wallis, Bonferroni-adjusted post hoc tests, chi-square/Fisher exact tests, MRI sensitivity analysis and ancillary achieved-power analysis are described. |
| Participants/results | Participant characteristics and cohort distributions reported in Table 1. |
| Descriptive data | Domain scores and item-level rates reported in tables and supplementary tables. |
| Outcome data | MRI and ultrasonography knowledge scores and misconception patterns reported. |
| Main results | Principal between-year comparisons and p values reported. |
| Other analyses | Sensitivity analysis for low-confidence MRI items described. |
| Key results | Discussion summarizes main findings. |
| Limitations | Single-centre, cross-sectional design; curriculum-specific external validity; unlinked samples; volunteer/selection bias; lack of independent expert-panel content validity; and answer-key limitations are discussed. |
| Interpretation | Interpretation linked to literature and educational implications. |
| Generalisability | Generalisability to dental schools with different curricula is explicitly limited; multicentre prospectively linked validation is recommended. |
| Funding | Funding statement included in Declarations. |
